# Employment for a Poor Half-Witted Girl

**Published:** 1887-01-22

**Authors:** 


### Difficult Cases.

[Inquiries and Cases are always welcome for this Column.]

### Employment for a Poor Half-witted Girl.

Miss L. Twining writes as follows in regard to this case:—(See The Hospital, p. 256).

"As no one has replied to the inquiry last week as to the disposal of the half-witted girl in a country workhouse, it may be of use (as such cases are numerous) if I state that there are now separate asylums for imbecile children and adults, in which they will receive all care and attention, and such instruction as they are capable of. Darenth is the great asylum for many of the London Unions for children; while Leavesden and Caterham are for adults. The clerk of every Union would know to which any case should be sent. But if the girl were not bad enough to be classed as an imbecile, such as she should be well cared for in her own workhouse, which seems to me to be the place intended for those who are destitute and incapable of earning their own living. There is plenty of work to be done in every workhouse, which a half-witted girl would be quite able to perform; and as 'able-bodied women' are now happily disappearing from the pauper ranks, such services would be thankfully utilised."

